# Editorial: Advances in the Prevention and Rehabilitation of Cardiovascular Diseases *via* Aerobic Exercise

**DOI:** 10.3389/fcvm.2022.858785

**Published:** 2022-03-14

**Authors:** Richard Y. Cao, Sebastian Kelle, Jian Yang

**Affiliations:** ^1^Department of Cardiology, Shanghai Xuhui Central Hospital, Fudan University, Shanghai, China; ^2^Department of Internal Medicine/Cardiology, Deutsches Herzzentrum Berlin, Berlin, Germany; ^3^German Centre for Cardiovascular Research (DZHK), Berlin, Germany; ^4^Department of Cardiology, Campus Virchow Klinikum, Charité—Universitätsmedizin Berlin, Freie Universität Berlin and Humboldt-Universität zu Berlin, Berlin, Germany; ^5^Department of Rehabilitation, Zhongshan-Xuhui Hospital, Fudan University, Shanghai, China

**Keywords:** cardiovascular disease, aerobic exercise, cardiac rehabilitation, exercise testing, mechanism

The 21st century is a new era for the rapid development of the cardiac rehabilitation programs. Publications in the field of cardiac rehabilitation research have grown greatly year by year in the past two decades, from <100 per year in 2001–2003 to over 600 in 2019 (Yuan et al.). Cardiac rehabilitation usually involves exercise training, psychological consultation, and lifestyle education to reduce cardiovascular risk factors. Growing evidence demonstrates that aerobic exercise-based cardiac rehabilitation plays an important role in the prevention and management of cardiovascular diseases and is therefore recommended by the American Association of Cardiovascular/Pulmonary Rehabilitation, American Heart Association, American College of Cardiology, as well as the European Society of Cardiology (1).

This Research Topic on “*Advances in the prevention and rehabilitation of cardiovascular diseases via aerobic exercise*” received an overwhelming number of submissions. The final collection brings together 12 original research articles, including five basic and seven clinical studies, three clinical trials containing one trial protocol and two trial reports, and eight review articles. This collection of research aims to reveal appropriate training protocols and evaluation methods to meet the requirements of both clinical and home-based rehabilitation programs. The articles collected under this Research Topic tend to produce high quality and timely articles involving advances in clinical applications and underlying molecular mechanisms from bench to bedside, and beyond, to highlight the underrepresented areas in controlling cardiac risk factors and preventing cardio-metabolic and cardiovascular diseases *via* exercise-based rehabilitation training. These articles represent exceptional research achievements in this field and contribute significantly to cardiovascular medicine and public health.

## Basic Science in Cardiac Rehabilitation

It is important to explore recent basic research in uncovering novel molecular mechanisms and identifying new theories related to cardiovascular pathophysiology in response to aerobic exercise. A research team tested cardiac-specific protein expression after a 4-week exercise using mass spectrometry-based proteomics in a mouse model of ischemia-induced heart failure (Mi et al.). Aerobic exercise-based rehabilitation training significantly up-regulated 597 proteins and down-regulated 707 proteins in comparison to those in the sedentary control group. In which, numerous proteins such as pyruvate dehydrogenase complex E1 component subunit alpha and subunit beta, pyruvate dehydrogenase kinase 2 and 4, and poly [ADP-ribose] polymerase 3 were associated with mitochondrial energy regulation, indicating that aerobic exercise might influence energy metabolism in heart failure mice. Another research team found that cardiac mitochondrial function in high aerobic capacity disease resistant phenotype was lost after ischemia-reperfusion insult in a rat model of coronary artery occlusion injury (Alsahly-a et al.). These studies propose potential molecular targets regarding mitochondrial regulation and function for future mechanism exploration. It was demonstrated that low capacity running rats had increased risk for ischemic injury characterized by high expression of vascular endothelial growth factor, endothelial nitric oxide synthase, and angiopoietin 2 after hind limb ischemia occlusion (Granier et al.). Therefore, high intrinsic aerobic capacity is essential for adaptive response to ischemic stress, though it may be influenced by gender differences (Alsahly-b et al.). Another study evaluated the influence of treadmill exercise on microglia polarization in a rat model of cerebral ischemia-reperfusion injury and found that exercise improved neurobehavioral outcomes, reduced infarct volumes, and increased interleukin-4 expression, which promoted M2 microglia activation along with the increasing of p-JAK1 and p-STAT6 expressions (Lu et al.). In a few words, the above-mentioned research demonstrates that aerobic exercise is beneficial for cardiac and cerebral vascular health, evidenced by using mice and rats as animal models.

## Clinical Practice in Cardiac Rehabilitation

International collaborations promote the development of the Physical Activity Readiness Questionnaire for Everyone (PAR-Q+) from a concept to an evidence-based document for safe engagement in the physical activity and fitness assessment (2). Now PAR-Q+ is the gold standard in clinical practice for pre-participation risk stratification and screening globally. The translation, cultural adaptation, and reproducibility of the new version of PAR-Q+ in the Brazilian Portuguese language may facilitate a large number of Brazilians to participate in safer physical activity (Schwartz, Oh, Takito et al.). The same research team undertook in a critical review that also interprets and synthesizes current approaches for the prevention of chronic conditions such as being overweight and obesity with regard to dietary guidelines and physical activity security, which will enable a healthier and more active lifestyle for people in Brazil and Canada (Schwartz, Oh, Perotto et al.).

Currently, cardiopulmonary exercise testing (CPET) is the gold standard for the measurement and evaluation of the cardiorespiratory system in response to stress during exercise but requires costly instruments and well-trained technicians. Some easy-to-handle methods such as the six-min walk test (6MWT) and treadmill exercise testing (TET) are used as alternative solutions to measure cardiopulmonary capacities. Thus, it may open a door to develop additional methods besides CPET for evaluating cardiopulmonary capacities before and after rehabilitation training. For instance, researchers identified determinants using 6MWT and generated two reference equations for predicting 6MWT distance in patients immediately after coronary artery bypass and valve surgery (Radi et al.). Moreover, the role of 6MWT guided by impedance cardiography during rehabilitation in patients with knee arthroplasty was proven to be effective in evaluating the recovery of lower limb function and the improvement of exercise capacity in a randomized controlled trial (Lin et al.). While other scientists showed that TET was also able to assess exercise capacity accurately besides CPET (Gao et al.). The maximum exercise capacity measured by TET was negatively correlated with the waist-hip ratio, implying that TET could represent physical status. Thus, in addition to CPET, measurements of 6MWT and TET are valuable methods for the evaluation of exercise capacity in clinical practice. However, body positions may alter measurements; for example, peak oxygen uptake in the upright position is higher than that in the supine position and such differences need further verification for clinical significance (Wan et al.). Furthermore, the effects of exercise training on cardiac structure, cardiac function, and cardiovascular risk profiles can also be characterized by imaging metrics, including parameters derived from echocardiography, magnetic resonance, and Doppler ultrasound (Alhumaid et al.).

In addition to evaluation methods and measurements, exercise intensity is also an important component in optimizing outcomes in cardiac rehabilitation. A research group reviewed and compared evidence for prescribing moderate-vigorous intensity aerobic exercise and high intensity interval training in cardiac rehabilitation programs and found that higher intensity exercise contributed to greater improvements in peak oxygen uptake than low-moderate intensity exercise; however, there was no one-size-fits-all model for high intensity interval training (Taylor J. L. et al.). The gradual introduction and progression of higher intensity exercise can maximize an individual's safety, adherence, and physiological outcomes. In addition, exercise prescription implementation rate may also have significant effects on cardiovascular outcomes (Zhu et al.).

Enhanced external counterpulsation is a non-invasive procedure that alleviates symptoms of angina and reduces myocardial ischemia in patients with coronary artery disease (3). A recent meta-analysis confirmed that enhanced external counterpulsation could improve exercise capacity in patients with chronic heart failure resulting from randomized controlled trials (4). An original study explored acute hemodynamic responses to enhanced external counterpulsation in patients with coronary artery disease. They found that enhanced external counterpulsation induced more significant acute responses of vascular and blood flow characteristics in carotid arteries than that in peripheral arteries with a gender difference, in which acute improvement of flow rate in the brachial artery was only shown in male participants and resistance index in carotid arteries was only changed in female participants (Zhang et al.). These results suggest that enhanced external counterpulsation can regulate the vascular and blood flow characteristics of conduit arteries and further ameliorate the carotid and probably peripheral vascular function in patients with coronary artery disease but the gender difference remains to be elucidated.

Heart failure, the terminal stage of several cardiovascular diseases with poor quality of life, is the major cause of morbidity and mortality globally (5). Exercise-based rehabilitation training has also been demonstrated to be suitable for heart failure patients. A group of scientists showed that high intensity interval training led to a similar inflammatory response as seen with traditional training in patients with chronic heart failure (Taylor A. G. et al.). A pilot randomized controlled trial of the *Baduanjin* Eight-Silken-Movements with Self-Efficacy was conducted in patients with chronic heart failure and proved to be feasible and effective (Chen et al.). Moreover, other clinician scientists designed a multicenter randomized controlled trial to test the efficacy and safety of different aerobic exercise intensities in heart failure patients with reduced ejection fraction (Shen et al.). The successful completion of this trial could potentially provide a basis for formulating appropriate exercise prescriptions for this patient population.

## Rehabilitation in Patients With Complicated Conditions

Exercise-based rehabilitation is beneficial for not only cardiovascular diseases but also other disorders. Low physical performance in patients with chronic kidney disease concomitant with heart failure was found to be associated with long-term mortality in a retrospective longitudinal study (Weng et al.). Moreover, the prevalence of chronic obstructive pulmonary disease coexistence in patients with chronic heart failure is challenging in developed countries with a large aging population because patients with coexistence of these two diseases present a further impaired exercise capacity, daily life activity, and health status. It showed that in addition to the pharmacological approach, cardiopulmonary rehabilitation, which could improve aerobic capacity, respiratory and peripheral muscle strength, was an optimized scheme to reduce hospital re-admission, morbidity, and mortality for these patients in comorbid chronic pulmonary disease and chronic heart failure (Borghi-Silva et al.). A systematic review and meta-analysis evaluated the structure and format of all kinds of exercise-based, multimodal rehabilitation programs used in individuals with cancer and showed that exercise-based rehabilitation could promote cardiopulmonary fitness, improve the cancer-specific quality of life, and reduce cardiovascular risk among cancer survivors (Rickard et al.). Moreover, another systematic review and meta-analysis supported the importance of exercise participation with both aerobic and resistance training in adults living with schizophrenia (Bredin et al.). Although disputed results of combination training exist in the above study, physical activity is still a real-world therapy to boost physical and psychological health for persons with schizophrenia, cancer survivors, and heart failure patients concomitant with chronic kidney disease or chronic obstructive pulmonary disease, and probably individuals with other chronic conditions who need ongoing healthcare and medical attention.

## Conclusions and Future Perspectives

The collection of articles in this Research Topic examines the effects of aerobic exercise in the prevention and rehabilitation of cardiovascular diseases with/without some concomitant chronic conditions. These studies elucidate not only the latest accomplishments in the field but also the unmet challenges in current clinical practice. Despite significant evidence of the benefits of exercise-based cardiac rehabilitation, controversial results of exercise still exist and key elements of exercise intensity, training duration, and evaluation methods remain to be illustrated. In addition, further understanding of the underlying molecular mechanisms of aerobic exercise in the prevention of cardiovascular diseases through basic science is desperately needed to optimize current rehabilitation programs. Since research has become a team sport more than ever before, we sincerely hope that researchers all over the world can work together to address these challenges and move the field of exercise-based cardiac rehabilitation forward through translational achievements in the near future.

## Author Contributions

RC drafted the editorial in consultation with JY and SK edited it. All authors approved it for publication.

## Funding

RC was supported by the National Natural Science Foundation of China (Grant number 81672260). JY was supported by Shanghai Municipal Health Commission (Grant number ZK 2019A10). SK received support from the DZHK (German Center for Cardiovascular Research). Partner Site Berlin was supported by an unrestricted research grant from Philips Healthcare.

## Conflict of Interest

The authors declare that the research was conducted in the absence of any commercial or financial relationships that could be construed as a potential conflict of interest.

## Publisher's Note

All claims expressed in this article are solely those of the authors and do not necessarily represent those of their affiliated organizations, or those of the publisher, the editors and the reviewers. Any product that may be evaluated in this article, or claim that may be made by its manufacturer, is not guaranteed or endorsed by the publisher.

## References

[1] American American Association of Cardiovascular and Pulmonary Rehabilitation; American College of Cardiology Foundation; American Heart Association Task Force on Performance Measures (Writing Committee to Develop Clinical Performance Measures for Cardiac Rehabilitation) Thomas RJ King M Lui K. AACVPR/ACCF/AHA 2010 update: performance measures on cardiac rehabilitation for referral to cardiac rehabilitation/secondary prevention services endorsed by the American College of Chest Physicians, the American College of Sports Medicine, the American Physical Therapy Association, the Canadian Association of Cardiac Rehabilitation, the Clinical Exercise Physiology Association, the European Association for Cardiovascular Prevention and Rehabilitation, the Inter- American Heart Foundation, the National Association of Clinical Nurse Specialists, the Preventive Cardiovascular Nurses Association, and the Society of Thoracic Surgeons. J Am Coll Cardiol. (2010) 56:1159–67. 10.1016/j.jacc.2010.06.006 20863958

[2] Warburton DER Gledhill N Jamnik VK Bredin SSD McKenzie DC Stone J. International launch of the PAR-Q+ and ePARmed-X. The physical activity readiness questionnaire (PAR-Q+) and electronic physical activity readiness medical examination (ePARmed-X+): summary of consensus panel recommendations. Health Fitness J Can. (2011) 4:26–37. 10.14288/hfjc.v4i2.103

[3] Bonetti PO Holmes DR Lerman A Barsness GW. Enhanced external counterpulsation for ischemic heart disease. J Am Coll Cardiol. (2003) 41:1918–25. 10.1016/S0735-1097(03)00428-5 12798558

[4] Zhou ZF Wang DJ Li XM Zhang CL Wu CY. Effects of enhanced external counterpulsation on exercise capacity and quality of life in patients with chronic heart failure: a meta-analysis. Medicine (Baltimore). (2021) 100:e26536. 10.1097/MD.0000000000026536 34232191 PMC8270628

[5] Groenewegen A Rutten FH Mosterd A Hoes AW. Epidemiology of heart failure. Eur J Heart Fail. (2020) 22:1342–56. 10.1002/ejhf.1858 32483830 PMC7540043

